# Childhood trauma is linked to epigenetic age deceleration in young adults with previous youth residential care placements

**DOI:** 10.1080/20008066.2024.2379144

**Published:** 2024-07-25

**Authors:** Maria Meier, Sina Kantelhardt, Laura Gurri, Christina Stadler, Marc Schmid, Vera Clemens, Aoife O’Donovan, Cyril Boonmann, David Bürgin, Eva Unternaehrer

**Affiliations:** a Child and Adolescent Psychiatric Research Department, University Psychiatric Clinics Basel (UPK), University of Basel, Basel, Switzerland; b Department of Psychology, University of Konstanz, Konstanz, Germany; c Department of Psychology, Friedrich-Schiller-University of Jena, Jena, Germany; d Department of Child and Adolescent Psychiatry/Psychotherapy, University of Ulm, Ulm, Germany; e Department of Psychiatry & Behavioral Sciences, University of California, San Francisco, CA, USA; f Mental Health Service, San Francisco Veterans Affairs Health Care System, San Francisco, CA, USA; g Department of Child and Adolescent Psychiatry (LUMC Curium), Leiden University Medical Center, Leiden, The Netherlands

**Keywords:** Childhood trauma, epigenetic aging, Horvath’s clock, Hannum’s clock, DNA methylation, early life stress, trauma, age deceleration, Trauma infantil, edad epigenética, metilación del ADN, estrés temprano, trauma, aceleración de la edad, reloj de Hannum, reloj de Horvath

## Abstract

**Background:** Early adversity increases the risk for mental and physical disorders as well as premature death. Epigenetic processes, and altered epigenetic aging in particular, might mediate these effects. While the literature that examined links between early adversity and epigenetic aging is growing, results have been heterogeneous.

**Objective:** In the current work, we explored the link between early adversity and epigenetic aging in a sample of formerly out-of-home placed young adults.

**Method:** A total of *N *= 117 young adults (32% women, age *mean *= 26.3 years, *SD *= 3.6 years) with previous youth residential care placements completed the Childhood Trauma Questionnaire (CTQ) and the Life Events Checklist (LEC-R) and provided blood samples for the analysis of DNA methylation using the Illumina Infinium MethylationEPIC BeadChip Microarray. Epigenetic age was estimated using Hovarth’s and Hannum’s epigenetic clocks. Furthermore, Hovarth’s and Hannum’s epigenetic age residuals were calculated as a proxy of epigenetic aging by regressing epigenetic age on chronological age. The statistical analysis plan was preregistered (https://osf.io/b9ev8).

**Results:** Childhood trauma (CTQ) was negatively associated with Hannum’s epigenetic age residuals, *β *= −.23, *p* = .004 when controlling for sex, BMI, smoking status and proportional white blood cell type estimates. This association was driven by experiences of physical neglect, *β *= −.25, *p* = .001. Lifetime trauma exposure (LEC-R) was not a significant predictor of epigenetic age residuals.

**Conclusion:** Childhood trauma, and physical neglect in particular, was associated with decelerated epigenetic aging in our sample. More studies focusing on formerly institutionalized at-risk populations are needed to better understand which factors affect stress-related adaptations following traumatic experiences.

## HIGHLIGHTS

- Growing literature links early adversity to altered epigenetic aging, yet results have been heterogeneous.
- We assessed childhood and lifetime trauma exposure using the Childhood Trauma Questionnaire and the Life Events Checklist and estimated epigenetic aging by obtaining Horvath’s and Hannum’s epigenetic age residuals in a sample of formerly out-of-home placed young adults.
- In this high-risk sample, childhood trauma, physical neglect in particular, but not lifetime trauma was negatively related to epigenetic aging.

## Abstract (es)

**Antedecentes.** La adversidad temprana incrementa el riesgo para trastornos físicos, mentales y, además, de muerte prematura. Los procesos epigenéticos, en particular procesos de senescencia epigenética alterada, podrían mediar estos efectos. Pese a que la literatura que evalúa la asociación entre adversidad temprana y edad epigenética es creciente, los resultados han sido heterogéneos.

**Objetivo.** En este trabajo se exploró la asociación entre adversidad temprana y senescencia epigenética en una muestra de adultos jóvenes con antecedentes de haber estado en hogares residenciales.

**Métodos.** Un total de *N* = 117 adultos jóvenes (32% mujeres, edad promedio = 26,3 años, desviación estándar = 3,6 años) con antecedente de haber sido asignados a hogares residenciales para jóvenes completaron el Cuestionario de Trauma Infantil (CTQ por sus siglas en inglés) y la Lista de Chequeo de Eventos Vitales (LEC-R por sus siglas en inglés) y brindaron muestras de sangre para el análisis de metilación del ADN empleando el microarray Infinitum MethylationEPIC de Illumina. La edad epigenética se estimó empleando los relojes epigenéticos de Hovarth y de Hannum. Además, los residuales de la edad epigenética de Hovarth y de Hannum se calcularon como un indicador indirecto de la edad epigenética al realizar una regresión de la edad epigenética con la edad cronológica. El plan para el análisis estadístico fue registrado con anterioridad (https://osf.io/b9ev8)

**Resultados.** El trauma infantil (CTQ) está inversamente asociado a los residuales de la edad epigenética de Hannum, *β* = −.23, *p* = .001 al controlar el sexo, el IMC, el tabaquismo y las estimaciones proporcionales del tipo de glóbulos blancos. Esta asociación fue impulsada por las experiencias de negligencia fisica, *β* = −.25, *p* = .001 La exposición al trauma a lo largo de la vida (LEC-R) no fue un predictor significativo de los residuales de la edad epigenética.

**Conclusión.** El trauma infantil, en particular la negligencia física, estuvo asociado a una ralentización en la edad epigenética en esta muestra. Se requieren más estudios enfocados en poblaciones con antecedentes de institucionalización para poder comprender mejor cuáles factores afectan los mecanismos de adaptación relacionados con el estrés luego de experiencias traumáticas.

## 1. Introduction

Childhood and adolescence are sensitive neurodevelopmental periods. Exposure to adverse, potentially traumatic events during that time, e.g. experiencing or witnessing domestic violence, or abuse and neglect by primary caregivers, can exert long-lasting health impairments (Luby et al., 2020). As such, early adversity increases the risk of illness and multimorbidity in adulthood (England-Mason et al., 2018), with higher rates of both mental (e.g. depression) and physical diseases (e.g. diabetes) being observed. In addition, early adversity is linked to a heightened risk of premature mortality (Lawrence et al., 2023; Rod et al., 2020; Yu et al., 2022). Thereby, a dose–response relationship is observed for the increased risk of both multimorbidity and mortality, as both risks increase with the number of adverse experiences (England-Mason et al., 2018). Overall, individuals with multiple early adverse experiences are at risk of dying approximately 20 years earlier than peers who have not experienced such events (Brown et al., 2009).

The heightened risks of disease and premature mortality may be due to dysregulations of multiple biological systems. On the neural level, profound alterations following early adversity have been found in the limbic circuits and related cortical structures (Chen & Baram, 2016; Hakamata et al., 2022; Pollok et al., 2022). Furthermore, systemic changes in the nervous, endocrine, inflammatory, cardiovascular, and metabolic systems have been observed (Cooke et al., 2023; Dich et al., 2015; Nemeroff, 2016). The structural and functional changes associated with early adversity are accompanied by behavioural impairments in related domains (Karatsoreos & McEwen, 2013). For example, altered fear/threat responses, reward learning, emotion regulation, and executive functions have been linked to early adversity (Callaghan & Tottenham, 2016; Pechtel & Pizzagalli, 2011). In conclusion, there is a growing body of evidence indicating that early adversity is associated with long-term impairments of multiple neurobiological systems and related behavioural outcomes.

These neuronal and systemic dysregulations might be mediated by altered neurobiological development, maturation and aging (McLaughlin et al., 2019). There are several theories – most prominently the stress acceleration hypothesis (Callaghan & Tottenham, 2016) – that propose that early adversity leads to premature maturation of emotion circuits (Ellis & Del Giudice, 2019). According to this theory, accelerated maturation enables the child to cope with emotions and stress independently of the unreliable caregiver. While this might result in adult-like behaviour in stress- and fear-related domains at an early age, it might come at cost of an overall impaired integrity of the emotional system long-term, with associated structural and functional pathology later in life (Callaghan & Tottenham, 2016; Miller, 2021). Similarly, the adaptive calibration model suggests that organisms adapt to their environment in a future-directed manner, thereby trying to enhance reproductive success and survival in the long term (Ellis & Del Giudice, 2019) – which has been supported by findings indicating earlier pubertal maturation following traumatic experiences (Binder et al., 2018; Hamlat et al., 2021; Hamlat et al., 2023). Following these theories, the adaptation to adverse environments might involve accelerated maturation processes at the cost of accelerated aging (Ellis & Del Giudice, 2019).

There are multiple *gene x environment* pathways that might contribute to accelerated development, including epigenetic processes that may be related to downstream alterations in neurobiological functioning and behaviour (Allis & Jenuwein, 2016; Gassen et al., 2017). Epigenetic processes comprise changes that impact gene expression without altering the genetic code per se. One of the most commonly studied epigenetic mechanisms is DNA methylation, which describes the attachment of a methyl group on the cytosine of cytosine–guanine dinucleotides (CpG sites) (Sadava et al., 2019). As the DNA methylation pattern changes with chronological age, it can be used to estimate the age of a given DNA string using machine learning algorithms (Ryan, 2021). These epigenetic clocks are said to ‘link developmental and maintenance processes to biological aging’ (Horvath & Raj, 2018) and thus reflect the age of an organ or tissue on a molecular level. If the estimated epigenetic age is higher than expected (operationalized by regressing epigenetic age on chronological age and obtaining epigenetic age residuals), this reflects accelerated epigenetic aging, which has been linked to mental and physical health status (Horvath & Raj, 2018; Ryan, 2021). In the context of early adversity, accelerated epigenetic aging may be associated with accelerated maturation processes that could open a gateway to heightened risk of disease and mortality.

However, previous studies linking early adversity to epigenetic aging provided inconsistent findings so far. While childhood trauma has been linked to accelerated epigenetic aging (Copeland et al., 2018; Jovanovic et al., 2017; Lawn et al., 2018; Sumner et al., 2019; Tang et al., 2020), several studies report null results (Etzel et al., 2022; Klopack et al., 2022; Lim et al., 2022). Likewise, post-traumatic stress disorder has been associated with both accelerated and decelerated epigenetic aging (Lim et al., 2022; Oblak et al., 2021). While high-risk groups of various kinds have been used to study the links between trauma exposure and epigenetic aging, studies focusing on formerly institutionalized individuals are lacking, as prior work in this population mostly focused on genome-wide DNA methylation patterns or DNA methylation of candidate genes (Kumsta et al., 2016; Naumova et al., 2012; Naumova et al., 2019; Non et al., 2016). Yet, (formerly) institutionalized individuals are known to be at particular risk for having been exposed to early adversity (Zelechoski et al., 2013) as well as for developing mental and physical health problems (Seker et al., 2022) which might be related to altered epigenetic aging.

We thus aimed to fill this gap by studying the links between trauma exposure, and epigenetic aging in a sample of previously out-of-home placed young adults. Using this sample, we previously found that early life adversity was linked to decelerated aging, as indexed by longer telomere length (Bürgin et al., 2022). Even though telomere aging processes and epigenetic aging processes seem to be unrelated to each other (Marioni et al., 2016; Raj & Horvath, 2020), we wanted to explore whether a similar pattern of decelerated aging occurs. In light of this, we planned to focus on first-generation epigenetic clocks that predict chronological age (Raj & Horvath, 2020), i.e. Horvath’s (Horvath, 2013) and Hannum’s epigenetic age (Hannum et al., 2013). Our preregistered analysis plan (https://osf.io/b9ev8) included two proof of concept analyses: We expected a significant positive linear correlation between epigenetic and chronological age in this high-risk sample, and we assumed that our sample demonstrates a significantly different epigenetic age compared with their chronological age. In our main hypothesis, we expected a significant association between early adversity and epigenetic aging, as operationalized using epigenetic age residuals. Since both, epigenetic age acceleration and deceleration after traumatic stress have been observed, and previous findings in the same sample showed an association between early adversity with longer telomere length (Bürgin et al., 2022), we tested our main hypothesis two-sided.

## 2. Methods

### 2.1. Sample and procedure

The data was collected from a sample of young adults with a history of residential youth care who took part in the Swiss Study for Clarification and Goal-Attainment in Child Welfare and Juvenile-Justice Institutions from 2007 to 2012 (German: Modellversuch Abklärung und Zielerreichung in stationären Massnahmen, MAZ.) (Schmid et al., 2013). In this study, *N* = 592 adolescents (*mean* age = 15.86 years, *SD* = 2.99 years, range = 5–27; 32.1% female) from 64 residential care institutions in Switzerland underwent a detailed clinical screening using both computer-based questionnaires (self- and external-assessment) and standardized clinical interviews at baseline (t0) and approximately one year later (t1).

Seven to twelve years later, a long-term follow-up study was conducted on those participants who agreed to be contacted again (*n* = 511). Overall, *n* = 203 subjects completed the questionnaires via the online platform WeAskYou (www.weaskyou.ch) (follow-up study: Youth welfare Trajectories: Learning from Experience, German: Jugendhilfeverläufe: Aus Erfahrung Lernen, JAEL) and were invited to a face-to-face visit including several (semi-structured clinical) interviews as well as a laboratory visit (t2) (Schmid et al., 2022).

While *n* = 185 participants participated in the on-site interviews, only a subset provided hair and blood samples for the analysis of neurobiological parameters (i.e. DNA methylation, hair cortisol concentrations, telomere length) (add-on studies: Long-Term Outcomes of Childhood Adversities and Offending Behavior, LOCO, and Long-term Outcomes of Childhood Adversities on DNA-Methylation, LOC-o-met). From the *n* = 131 participants who provided blood samples, *n* = 117 gave consent for further use of samples and data beyond the scope of the LOCO study’s objectives.

Therefore, the presented, cross-sectional analyses focusing on t2 are based on the data of *n* = 117 participants (32.5% women) with a mean age of 26.3 years (*SD* = 3.58 years; range = 16–38). Most participants were born in Switzerland (79.49%) or other European countries (9.40%). A participant flow chart can be found in Appendix Figure S1.

All participants provided written informed consent and received vouchers of up to CHF 500 as reimbursement for participation in the online assessments, the face-to-face interviews and the blood and hair sampling. The study was carried out in accordance with the Declaration of Helsinki and was approved by the Ethics Commission of Northwestern Switzerland (EKNZ, Ref. 2017-00718; project titles: MAZ., JAEL, LOCO and LOC-o-met).

### 2.2. Measures

***Childhood trauma exposure.*** Early adversity was assessed as a retrospective self-report at young adult age (t2) using the short form of the Childhood Trauma Questionnaire (CTQ) (Bader et al., 2009; Paquette et al., 2004). The CTQ assesses trauma exposure across childhood and adolescence with 28 items that are rated on a 5-point Likert scale. Of note, some of the CTQ items are collected with a prompt to reflect family exposures. For a population with history of institutionalization, these exposures may reflect experiences before placement, concurrent with placement such as during visits, or upon reunification. In our statistical analyses, we used the sum score of all CTQ items (excluding the three items of the *trivialization/denial* scale, theoretical range of CTQ score: 25–125) as a measure of *childhood trauma exposure*. In the exploratory analysis, we furthermore used the five subscales of the CTQ, including the scales emotional neglect and abuse, as well as physical neglect and abuse and sexual abuse.

***Lifetime trauma exposure*** The revised Life Events Checklist Revised (LEC-R) (Gray et al., 2004) was used to measure *lifetime trauma exposure* at young adult age (t2). The LEC-R assesses the exposure to potentially traumatic events by indexing which of 18 events participants have either experienced or witnessed during their lifetime, while allowing to rate one additional event that might not have been covered by the given items. In our statistical analysis, we used the number of potentially traumatic events that participants reported having experienced or witnessed as an index of the number of traumatic exposures (theoretical range of LEC-R score: 0–19) as a measure of *lifetime trauma exposure*.

***Chronological age.*** The chronological age variable used in the analysis reflects age at the time of the blood draw (t2) which was calculated as difference in years between t2 and participants’ birth date. We refer to this variable as *chronological age*.

***Epigenetic age.*** Epigenetic age at t2 was estimated based on DNA methylation that was determined from whole blood using the Illumina Infinium® MethylationEPIC BeadChip (Illumina Inc, San Diego, CA, USA) at the Human Genomics Facility (HuGe-F) Rotterdam, the Netherlands. Samples were drawn between 9 and 11 am and subsequently stored at −80°C until shipped and analyzed. We preprocessed the EPIC methylation data (idat files) in R version 4.2.0 using the *minfi* package (Aryee et al., 2014) according to recommendation (Fortin et al., 2017) and calculated Horvath’s (Horvath, 2013) and Hannum’s epigenetic clocks (Hannum et al., 2013) as estimators of epigenetic age using the command *agep()* of the *wateRmelon* package (Pidsley et al., 2013). We refer to these variables as *Hannum’s and Horvath’s (epigenetic) age*.

***Epigenetic age residuals.*** Epigenetic age residuals were obtained by regressing estimated epigenetic age (for Horvath’s and Hannum’s age estimates) on chronological age. We extended the approach by using a subtraction-based index (subtracting chronological age from estimated epigenetic age) (Ryan, 2021). In both indices, positive values indicate that the epigenetic age was higher than the chronological age (epigenetic age acceleration), and negative values indicate that epigenetic age was lower than the chronological age (epigenetic age deceleration) (Horvath & Raj, 2018; Simpson & Chandra, 2021). We refer to these variables as *Hannum’s and Horvath’s (epigenetic) age residuals*. Since the residual-based values are most commonly reported, we report results obtained from this approach in the manuscript. The results of the distance-based index can be retrieved from the html output file in the Appendix.

***Potential confounders.*** In our preregistration, we considered sex (operationalized as self-reported sex assigned at birth [male/female]) (Bath, 2020; Kankaanpää et al., 2022), socio-economic status in childhood and adolescence (operationalized as reporting financial problems of the family of origin at the baseline MAZ. assessment [yes/no]) (Fiorito et al., 2017; Walsh et al., 2019) and migration background (operationalized as either the participant or one of the parents were born outside of Switzerland [yes/no]) (Liel et al., 2020) as potential confounders, as these variables have been shown to be associated with early adversity as well as epigenetic age residuals in previous work. Body mass index (BMI) was assessed via self-reported height and weight at t2. Smoking status was predicted from the methylation data using the command *epismoker() (*Bollepalli et al., 2019). The control for proportional white blood cell types present in the sample (Teschendorff et al., 2017) we estimated CD4 + and CD8+ T-cells (CD4 T and CD8 T), natural killer cells (NK), monocytes (Mono) and b-cells (Bcell) using the command *estimateCellCounts()* of the *minfi* package *(*Aryee et al., 2014).

***Potential mediators.*** In an exploratory manner, we investigated potential mediators of the link between early adversity and epigenetic aging, namely lifestyle factors like smoking status (McCrory et al., 2022) and BMI (Yang et al., 2022), as well as current psychopathology (operationalized as internalizing and externalizing problems (Copeland et al., 2022) as indexed using subscales of the Achenbach System of Empirically Based Assessment [ASEBA]) (Achenbach et al., 2005, 2008). These variables have been associated with both, early adversity as well as DNA methylation in previous studies. Except for predicted smoking status, which was estimated from methylation data, all potential mediators were assessed via self-report at t2. Furthermore, we considered hair cortisol at t2, an index of chronic hypothalamus-pituitary-adrenal (HPA) axis activation, the major stress axis that has been previously discussed to be closely intertwined with aging processes (Pardon, 2007), as a potential mediator. More details on how this marker was assessed and analyzed can be found in previous publications (Bürgin et al., 2022).

### 2.3. Statistical analysis

We preregistered our hypotheses and analysis plan on November 30, 2022 (https://osf.io/b9ev8). At this time point, the data were already collected, and several findings, including findings on early adversity, telomere length, and hair cortisol concentration, had been published (Bürgin et al., 2022; Bürgin et al., 2022; d’Huart et al., 2022; Seker et al., 2022). However, neither descriptive statistics nor confirmatory statistical analyses had been computed on the DNA methylation data.

As the missing completely at random (MCAR) test (Little, 1988) indicated that missing values were missing completely at random and at least 50% of the data was available for all respective variables, we imputed missing values in predictors, mediators and covariates on item level to generate ten imputed data files using Predictive Mean Matching (PMM). There were no missing values in the CTQ and one missing value in the LEC-R.

First, as a proof of concept analysis, we used Pearson’s correlation to test for a correlation between epigenetic age (Horvath’s and Hannum’s age) and chronological age. As the normal distribution of chronological and epigenetic age variables was not met, we additionally computed Spearman’s rank correlation. Next, we used two-sided, paired (one-sample) t-tests to test whether participant’s epigenetic age and chronological age differed significantly from each other.

Second, to test our main hypothesis, we used linear regression models to predict epigenetic age residuals from childhood trauma exposure (CTQ) and lifetime trauma exposure (LEC-R) and pooled the results of all imputed datasets. Before running the analyses, assumptions for linear regressions (e.g. linearity of effect) were tested. To account for four statistical models used to test this hypothesis, using two operationalizations for traumatization (CTQ and LEC) as well as two measures of epigenetic age residuals (Horvath and Hannum), we Bonferroni adjusted the α level to α = .0125 in these analyses. We checked the models for outliers using the Bonferroni Outlier Test and for influential cases using Cook’s distance (Fox & Weisberg, 2019); no outliers or influential cases were detected. As sensitivity analysis, we preregistered that we would adjust for potential confounders (sex, socio-economic status, migration background) if they significantly correlated with the CTQ (subscales), the LEC-R score, or epigenetic age residuals. To address comparability between analyses of different studies and acknowledge that proportional white blood cell types need to be controlled for, we included sex, BMI, smoking status, and proportional white blood cell type estimates of CD4 T, CD8 T, NK, Mono, and Bcell as covariates in our final models.

Third, if CTQ total score was a significant predictor of epigenetic age residuals, we planned to conduct further exploratory analyses to differentiate between the impact of different categories of trauma. Therefore, we additionally ran our analyses using the subscales of the CTQ (i.e. physical neglect, physical abuse, emotional neglect, emotional abuse, sexual abuse) as predictors in multiple linear regression models.

Fourth, to explore the role of variables that potentially mediate the link between childhood trauma and epigenetic age residuals (cf. potential mediators), we confirmed mutual associations between trauma measures (the predictor) and epigenetic age residuals (the outcome) with the potential mediators using Pearson’s and Spearman’s correlations, as significant a and b paths are a prerequisite of a mediation analysis (Baron & Kenny, n.d.). If significant relationships could be confirmed, we ran bootstrapped mediation models (number of iterations = 10,000).

Last, we explored the associations between epigenetic age residuals and telomere length using Pearson’s and Spearman’s correlations. To control telomere length for the effect of chronological age, we regressed telomere length on chronological age and calculated the correlations using the obtained residuals. All exploratory analyses were only conducted with epigenetic age residuals that were significantly related to trauma in the primary analysis.

At the time of registration, we calculated a sensitivity power analysis to estimate the least detectable effect size using G*Power (Faul et al., 2007). Based on our sample size of *N* = 117, we could detect a Pearson’s correlation of *r* > =  .289 and an effect size of *d* > =  .300 in the paired t-test in our proof of concept analyses. In the basic and confounder-adjusted regression models that were preregistered to test our main hypothesis, we could detect an effect size of *f^2^* = .091.

All statistical analyses were performed in R version 4.3.0 and in RStudio version 2023.3.0.386 using the packages *car* (Fox & Weisberg, 2019), *corrplot* (Wei & Simko, 2021), *dplyr* (Wickham et al., 2023), *haven* (Wickham et al., 2023), *Hmisc* (Harrell, 2023), *lm.beta* (Behrendt, 2023), *mediation (*Tingley et al., 2014)*, mice* (Buuren & Groothuis-Oudshoorn, 2011), *naniar* (Tierney & Cook, 2023), and *reshape2* (Wickham, 2007). Figures were created with *ggplot2* (Wickham, 2016) and *patchwork* (Pedersen 2019).

## 3. Results

### 3.1. Preliminary and proof of concept analyses

Descriptive statistics of the current sample can be retrieved from Table 1. Participants included in this analysis did not differ from the rest of the sample regarding demographic variables, traumatization, internalizing or externalizing symptoms at baseline or at the 10-year follow-up assessment (see Appendix Table S1).

**Table 1.** Description of the sample.

Variable	Sample (*n* = 117)
Age at blood draw at t2 (in years)	26.3 (3.58)
Sex (self-reported) in # [female / male]	38 / 79
Migration background in # [yes / no] ^a^	66 / 51
Age at baseline in years	15.95 (3.00)
Age at first placement in years	11.33 (4.91)
Number of placements	3.99 (4.09)
Reason for out-of-home placement in % [civil / criminal / other]	48.72 / 22.22 / 23.93
PTSD diagnosis at t2 (SCID-5 interview) [no (%) / yes (%)]	111 (94.87%) / 6 (5.13%)

Note*.* If not otherwise stated, numbers represent mean (SD). *^a^* operationalized as either one parent or the participant was born outside of Switzerland.

According to previously established severity classifications (Witt et al., 2017), 77.78% of the sample reported at least moderate to severe experiences in at least one of the subscales of the CTQ (see Appendix Figure S2). On average, participants stated to have experienced or witnessed 4.66 potentially traumatic events (LEC-R; *SD* = 2.84, range: 0 to 12, missing = 1). Merely 14.66% of participants reported to have experienced or witnessed no or one potentially traumatic event. We observed a moderate positive correlation between *childhood trauma exposure* (CTQ) and *lifetime trauma exposure* (LEC-R), *r*(114) = .37, *p* < .001. The epigenetic age estimates, Horvath’s and Hannum’s age, showed a strong positive correlation, *r*(115) = .71, *p* < .001.

Sex was associated with *childhood trauma exposure* (CTQ), *r*(115) = .36, *p* < .001, but not with *lifetime trauma exposure* (LEC-R), *r*(114) = .12, *p* = .192. Specifically, female participants reported significantly higher numbers of *childhood trauma exposure* (CTQ) compared to male participants, *t*(65.38) = 3.99, *p* < .001, *d *= 0.82. Neither socio-economic status nor migration background were significantly associated with *childhood trauma exposure* or *lifetime trauma exposure* (see correlation matrix in Appendix Figure S3).

*Childhood trauma exposure* did not significantly differ between the groups with different placement reasons (civil law: *mean* = 53.37, *SD* = 15.96; penal law: *mean *= 48.35, *SD* = 12.01; other reasons: *mean *= 53.75, *SD* = 17.65), *F*(1,109) = 0.02, *p* = .881, partial η^2^ < .01. Likewise, *lifetime trauma exposure* did not significantly differ between the groups with different placement reasons (civil law: *mean* = 4.56 events, *SD* = 2.92, range: 0 to 12; penal law: *mean *= 4.76 events, *SD* = 2.82, range: 0 to 10; other reasons: *mean *= 4.96 events, *SD* = 2.82, range: 0 to 10), *F*(1,109) = 0.39, *p* = .536, partial η^2^ < .01. Furthermore, groups with different placement reasons did not differ regarding the extent of reported *internalizing problems* (ASEBA; civil law: *mean* = 54.07, *SD* = 11.29, range: 34 to 78; penal law: *mean *= 48.42, *SD* = 7.97, range: 29 to 62; other reasons: *mean *= 56.14, *SD* = 10.9, range: 34 to 79), *F*(1,109) = 0.13, *p* = .716, partial η^2^ < .01, or *externalizing problems* (ASEBA; civil law: *mean* = 54.63, *SD* = 9.92, range: 36 to 91; penal law: *mean *= 48.46, *SD* = 7.64, range: 31 to 66; other reasons: *mean *= 55.36, *SD* = 8.77, range: 36 to 74), *F*(1,109) = 0.04, *p* = .846, partial η^2^ < .01.

Chronological age showed a strong positive Pearson’s correlation with Horvath’s age, *r*(115) = .70, *p *< .001 (Spearman’s rank correlation: *r*(115) = .54, *p* < .001), and Hannum’s age, *r*(115) = .72, *p* < .001 (Spearman’s rank correlation: *r*(115) = .65, *p* < .001). The scatterplots illustrating the linear association can be found in Appendix Figure S4. Both epigenetic age estimates differed significantly from participants’ chronological age. While Horvath’s clock overestimated participants’ age on average by 2.6 years (mean Horvath’s age = 28.94), *t*(116) = −8.12, *p* < .001, *d* = −0.75, Hannum’s clock underestimated participants’ age on average by 6.6 years (*mean* Hannum’s age = 19.71), *t*(116) = 25.24, *p* < .001, *d* = 2.33 (see Appendix Figure S5).

### 3.2. Link between early adversity and epigenetic age residuals

**Figure 1.**
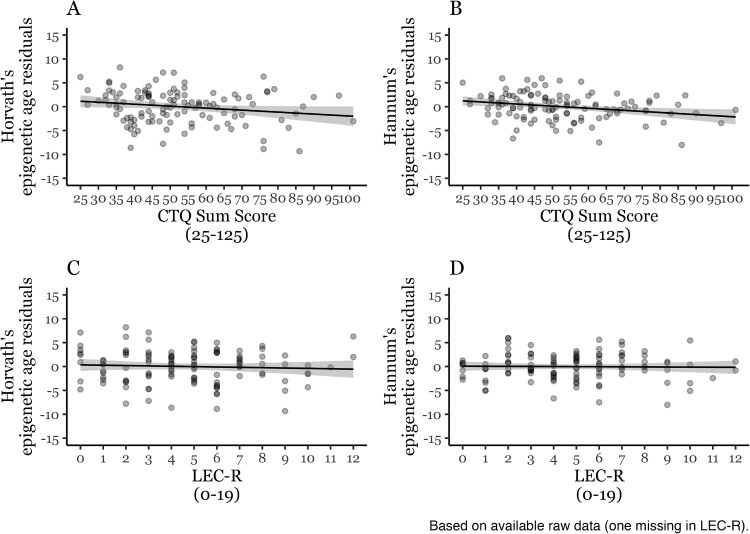
Linear relationship of childhood trauma exposure (A, B; *n* = 117) and lifetime trauma exposure (C, D; *n* = 116) and Horvath’s (A, C), and Hannum’s epigenetic age residuals (B, D). CTQ = Childhood Trauma Questionnaire. LEC-R = Life Events Checklist Revised.

*Childhood trauma* (CTQ, Table 2), but not *lifetime trauma exposure* (LEC-R, Table 3), showed negative associations with epigenetic age residuals when controlling for sex, BMI, smoking status, and proportional white blood cell type estimates (CD8 T, CD4 T, NK, Mono, Bcell), but only the effect of *childhood trauma exposure* on Hannum’s epigenetic age residuals survived the α level adjustment for four analyses (α = .0125). The related scatterplots are depicted in Figure 1. When excluding participants with a current PTSD diagnosis (*n* = 6), the results remained unchanged (cf. html output in the Appendix). The results of the preregistered models showed the same patterns and are provided in Appendix Tables S2 and S3.

**Table 2.** Parameters of the regression models predicting epigenetic age residuals from childhood trauma exposure (CTQ) controlled for sex (1 = male, 2 = female), body mass index (BMI), smoking status (1 = current smoker, 2 = never smoker), and proportional white blood cell type estimates. The estimates were pooled across ten imputed datasets. *P*-values marked with an asterisk survived the α level adjustment for four analyses (α = .0125).

	β	SE	*p*-value
***Horvath’s epigenetic age residuals***			
Intercept	−.19	.31	.535
CTQ	−.18	.10	.060
Sex	.05	.22	.813
BMI	−.01	.10	.927
Smoking status	.33	.21	.108
T helper cell CD8+ (CD8 T)	.18	.10	.082
T helper cell CD4+ (CD4 T)	−.03	.10	.787
Natural killer cells	.19	.10	.051
B-cells	−.11	.10	.250
Monocytes	−.06	.10	.575
***Hannum’s epigenetic age residuals***			
Intercept	−.59	.25	.022
CTQ	−.23	.08	.004*
Sex	.44	.18	.014
BMI	.08	.10	.412
Smoking status	.01	.17	.992
T helper cell CD8+ (CD8 T)	−.31	.08	.001*
T helper cell CD4+ (CD4 T)	−.33	.08	.001*
Natural killer cells	.27	.08	.001*
B-cells	.07	.08	.403
Monocytes	−.01	.08	.996

**Table 3.** Parameters of the regression models predicting epigenetic age residuals from lifetime trauma exposure (LEC-R) controlled for sex, BMI, smoking status, and proportional white blood cell type estimates (imputed data). The estimates were pooled across ten imputed datasets. *P*-values marked with an asterisk survived the α level adjustment for four analyses (α = .0125).

	β	SE	*p*-value
***Horvath’s epigenetic age residuals***			
Intercept	.01	.30	.987
LEC-R	−.03	.09	.780
Sex	−.09	.21	.654
BMI	−.01	.11	.902
Smoking status	.32	.21	.133
T helper cell CD8+ (CD8 T)	.18	.10	.078
T helper cell CD4+ (CD4 T)	−.05	.10	.618
Natural killer cells	.21	.10	.036
B-cells	−.13	.10	.178
Monocytes	−.07	.10	.504
***Hannum’s epigenetic age residuals***			
Intercept	−.32	.25	.194
LEC-R	.01	.08	.989
Sex	.25	.17	.154
BMI	.08	.10	.452
Smoking status	−.01	.17	.934
T helper cell CD8+ (CD8 T)	−.31	.08	.001*
T helper cell CD4+ (CD4 T)	−.36	.07	.001*
Natural killer cells	.29	.08	.001*
B-cells	.04	.08	.657
Monocytes	−.02	.09	.806

### 3.3. Exploratory analyses

As Horvath’s epigenetic age residuals did not survive the α correction in our primary analysis, we conducted the exploratory analysis only using Hannum’s epigenetic age residuals.

**Link between different categories of childhood trauma and epigenetic age residuals.** To account for five individual models, using five childhood trauma categories (physical neglect, emotional neglect, physical abuse, emotional abuse, sexual abuse) times one epigenetic age residual measure, we adjusted the α level to α = .01. Only physical neglect was a significant predictor of Hannum’s epigenetic age residuals, *β *= −.25, *p* = .001, when controlling for sex, BMI, smoking status, and proportional white blood cell type estimates. For the other subscales, we found some patterns that did not survive the multiple testing correction. Specifically, Hannum’s epigenetic age residuals were associated with emotional neglect, *β *= −.18, *p* = .019, and emotional abuse, *β *= −.18, *p* = .026. We found no associations for the sexual and physical abuse subscales. The detailed results of the regression models can be retrieved from the html output file in the Appendix.

**Impact of potential mediators.** We found significant Pearson’s (and Spearman’s) correlations between *childhood trauma exposure* and externalizing problems (A path), *r*(115) = .33, *p *< .001 (Spearman: *r*(115) = .28, *p *= .003); further, externalizing problems were significantly associated with Hannum’s epigenetic age residuals (B path), *r*(115) = −.23, *p *= .014 (Spearman: *r*(115) = −.23, *p *= .013). As such, externalizing symptoms fulfilled the criteria for a mediation analysis.

We thus calculated a bootstrapped mediation model with externalizing problems as the mediator between *childhood trauma exposure* and Hannum’s epigenetic age residuals, while controlling for sex, BMI, smoking status, and proportional white blood cell type estimates. The bootstrapped mediation analysis showed that there was a significant total effect from childhood trauma exposure to epigenetic age residuals, *estimate* = −.04, *p* = .002 (95% CI lower: −0.07; upper: −0.02). The average direct effect (ADE) from childhood trauma exposure to epigenetic age residuals remained significant when adding externalizing problems to the model, *estimate* = −.04, *p* = .020 (95% CI lower: −0.07; upper: −0.01). However, the average causal mediation effect (ACME) was not significant, *p *> .05, indicating that externalizing problems did not mediate the link between *childhood trauma exposure* and Hannum’s epigenetic age residuals.

None of the other potential mediators (i.e. internalizing problems, smoking status, BMI, hair cortisol levels) showed consistent *mutual* associations with childhood trauma and Hannum’s epigenetic age residuals (see correlation matrix in Appendix Figure S6 and S7).

**Link between epigenetic aging markers and telomere length.** We found no significant associations between epigenetic age residuals and age-adjusted telomere length (all *p *> .05; see correlation matrix in Appendix Figure S8).

## 4. Discussion

This study examined the links between early adversity and epigenetic aging in a sample of previously out-of-home placed, young adults. We found that childhood trauma – but not lifetime trauma exposure – was negatively related to epigenetic aging. Even though this trend was consistent across all analyses, only the negative association between childhood trauma exposure and Hannum’s epigenetic age residuals survived the correction for multiple testing. The finding contradicts previous theories (e.g. the stress acceleration theory, life history theory) (Callaghan & Tottenham, 2016; Gassen et al., 2017) and findings of accelerated epigenetic aging following early adversity (Copeland et al., 2022; Joshi et al., 2023; Jovanovic et al., 2017; Klopack et al., 2022; Lim et al., 2022; Wolf et al., 2018; Yang et al., 2022). While decelerated epigenetic aging has been reported in patients with PTSD (Oblak et al., 2021), the point prevalence of current PTSD in our sample was relatively low (5.13%) (Seker et al., 2022) and excluding individuals with current PTSD diagnosis did not affect the interpretation of the results.

Complementing these findings, our exploratory analyses revealed that the negative association between childhood trauma and epigenetic aging seemed to be driven by the experience of physical neglect specifically. Previous findings suggested that – in contrast to threat-related experiences which were linked to *accelerated* epigenetic aging – deprivation-related experiences, such as neglect, were *not* associated with accelerated epigenetic aging (Hamlat et al., 2023;Sumner et al., 2019; Colich et al., 2020; Rampersaud et al., 2022). Instead, neglectful experiences might contribute to postponing maturation processes until favourable conditions arise, which might explain the links to *decelerated* aging (Colich et al., 2020; Johnson et al., 2018; Rampersaud et al., 2022) as found in our study. Of note, physical neglect is among the most prevalent forms of childhood maltreatment in our sample (cf. Appendix Figure S2), which might partly explain the domain-specific and decelerating effects of childhood maltreatment we observed. Interestingly, early adversity was related to longer telomere length in the same high-risk sample in previous reports (Bürgin et al., 2022; Bürgin et al., 2022). This is noteworthy, as telomere length is an aging marker not associated with epigenetic aging (Marioni et al., 2016; Raj & Horvath, 2020) – a finding also supported in our analysis. As such, childhood trauma seems to be associated with decelerating effects on both aging processes examined in this specific cohort of formerly institutionalized young adults. While the majority of our sample reported at least some degree of early adversity, the domain-specific associations might be specific to our heterogeneous sample. As such, it is questionable whether the links observed here are generalizable to other at-risk populations with a history of institutionalization. To the best of our knowledge, there are no other studies on epigenetic aging in formerly institutionalized individuals, as most previous reports in this population focused on genome-wide DNA methylation patterns or DNA methylation of candidate genes (Kumsta et al., 2016; Naumova et al., 2012; Naumova et al., 2019; Non et al., 2016). Future studies in other formerly institutionalized cohorts thus have to determine the replicability of our findings.

Some limitations should be considered when interpreting our results. First, our sample was relatively small, diminishing the statistical power to detect small effects that are commonly reported in the epigenetic aging literature (Wolf et al., 2018) and run subgroup analyses. The sample is furthermore characterized by experiences of neglect in particular, which might have critically affected the results. Second, this study exclusively focused on individuals with a history of out-of-home placements, which is thus intertwined with the effects of early adversity. Further, as epigenetic alterations are reversible, (therapeutic) interventions might have modulated the results (even though DNA methylation changes were only weakly associated with therapy outcomes in prior studies) (Hummel et al., 2022). Lastly, epigenetic aging, childhood and lifetime trauma exposure were (on average) assessed in participant’s mid-twenties, yet some associations might only be detectable on a larger time scale. Although previous reports have also reported null results concerning lifetime trauma exposure (Wolf et al., 2018), the fact that it was assessed in early adulthood might influence our results. Thus, future studies should expand the measurement period to further explore the effects of trauma exposure on epigenetic aging beyond young adult age. Despite the big temporal overlap, childhood and lifetime trauma exposure were only weakly associated with each other. This might be related to the low content overlap of both scales, i.e. the fact that the CTQ focuses on interpersonal stressors primarily within the family context, while the LEC-R puts more weight on external stressors like natural disasters (Koppold et al., 2023). As individuals were, on average, removed from their family of origin at age 11, the interpretation of the CTQ scales might be restricted in this cohort as opposed to non-institutionalized samples. Yet, as many participants were still in contact with their family during their stays in the placements and/or visited their family on the weekends, we deem that participants likely disclosed experiences made within their family of origin when filling in the CTQ. Overall, this might imply that interpersonal stressors – especially when experienced in the caregiver context (Opendak et al., 2017) – seem to be particularly relevant, highlighting the importance of primary caregivers as potential source and buffer against adverse experiences in early life (Unternaehrer et al., 2021).

In sum, we found that greater exposure to childhood trauma – especially physical neglect – was linked to Hannum’s epigenetic age deceleration in a high-risk sample of previously out-of-home placed young adults. Our results challenge the notion that associations across the early adversity spectrum, across different aging markers and different adversity subtypes consistently line up. Similarly to the heterogeneous findings regarding the links between early adversity and epigenetic aging processes, the reported links between early adversity (Bunea et al., 2017; DeSantis et al., 2011; Rao et al., 2008), PTSD (Klaassens et al., 2012; Maercker et al., 2022; Speer et al., 2019) and HPA axis regulation are rather heterogeneous as well, reporting hyper-, hyporegulation, as well as no alteration. It can be assumed that these discrepancies are related to the complex neurobiological changes that have been linked to trauma (Agorastos & Chrousos, 2022). However, further research is needed to elucidate which factors (e.g. trauma-related variables such as type, timing and duration) play a modulating role in this context.

## CRediT author statement

Maria Meier: Formal analysis, Visualization, Writing – Original Draft, Writing – Review & Editing

Sina Kantelhardt: Formal analysis, Visualization, Writing – Review & Editing

Laura Gurri: Data Curation, Writing – Review & Editing

Christina Stadler: Resources, Writing – Review & Editing

Marc Schmid: Resources, Writing – Review & Editing, Funding acquisition

Vera Clemens: Writing – Review & Editing

Aoife O’Donovan: Writing – Review & Editing

Cyril Boonmann: Writing – Review & Editing

David Bürgin: Investigation, Data Curation, Writing – Review & Editing, Funding acquisition

Eva Unternaehrer: Formal analysis, Writing – Review & Editing

## Supplementary Material

AgeAccel_ReviewerResponse_20240628.pdf

## Data Availability

Data are available upon reasonable request.
