# Supplementary material for: Childhood trauma is linked to epigenetic age deceleration in young adults with previous youth residential care placements
Source: Eur J Psychotraumatol. 2024 Jul 25;15(1):2379144. doi: 10.1080/20008066.2024.2379144 (PMC11275517; doi:10.1080/20008066.2024.2379144)
Supplement: AgeAccel_ReviewerResponse_20240628.pdf [file ZEPT_A_2379144_SM4507.pdf]

Comments from the Editors and Reviewers:

Thank you for taking the time to review the latest version of our manuscript. Please find our responses to the individual points below. We have revised the manuscript and hope that we have addressed all of your concerns to your complete satisfaction.

Reviewer #1:

This revised manuscript has been highly responsive to the prior round of reviews and I think will make a ni
ce contribution to the literature. I have only minor concerns that should be easily addressed, as outlined below.

1). Please carefully review the entire manuscript for causal language and remove it. For example, on page 6, line 102, calling epigenetic aging a "byproduct" of stress and on line 109, referring to the "effects" of trauma on epigenetic aging is not supported by this study or any prior study that I'm aware of. Please revise with phrases more appropriate for associations/correlations.

We have carefully revisited the entire manuscript and rephrased any expressions that might imply causality (e.g. effects, predicted, etc.). For example, we rephrased the “byproduct sentence” to “In the context of early adversity, accelerated epigenetic aging may be associated with accelerated maturation processes that could open a gateway to heightened risk of disease and mortality.” (line 157). Further, we speak of “links between trauma and epigenetic aging” throughout the manuscript.

2). Page 11, line 260—the use of "by" in the sentence "age residuals by childhood trauma exposure…" is confusing as "by" is often used to describe moderation. Instead, I would rephrase as "from."

We have rephrased the sentence which now reads “we used linear regression models to predict epigenetic age residuals from childhood trauma exposure (CTQ) and lifetime trauma exposure (LEC-R) and pooled the results of all imputed datasets” (line 325).

3). In the prior review, I had suggested using the CD4T, CD8T, NK, Mono, and Bcell estimates from one of two scripts as covariates in the analyses and I greatly appreciate that the authors did this. I had intentionally left granulocytes off that list for the reasons that the authors identified—collinearity with the other cell types. This is not an error, but rather embedded in the calculation of those cell types so we always omit them from the covariates. These are proportional white blood cell types so there is an inherent dependency between them. Given this, and to avoid confusion, I would simply omit discussion of the granulocytes and never test them in the model (so don't need to get into the high VIF, etc). As well, please clarify throughout the manuscript that these are proportional white blood cell type estimates.

Thanks for the clarification. We have removed the information about Gran from the text and have clarified throughout the mansucript that we used proportional white blood cell type estimates.

4). On page 12 of the PDF, line 310, I would not refer to these as "confirmatory" analyses. I am not sure what is confirmatory about them. Maybe these are the "primary" analyses?

We have changed the sentence to: “All exploratory analyses were only conducted with epigenetic age residuals that were significantly related to trauma in the primary analysis.” (line 356).

5). On page 13, line 340, "degree" does not seem to be the correct term. What about "number" instead?

We have changes the sentence according to your suggestion which now reads “female participants reported significantly higher numbers of *childhood trauma exposure* (CTQ) compared to male participants”. (line 400).

We thank the reviewer for their constructive feedback.
